# Characterization of the Biosynthetic Gene Cluster of Enterocin F4-9, a Glycosylated Bacteriocin

**DOI:** 10.3390/microorganisms9112276

**Published:** 2021-11-01

**Authors:** Mohamed Abdelfattah Maky, Naoki Ishibashi, Jiro Nakayama, Takeshi Zendo

**Affiliations:** 1 Department of Food Hygiene and Control, Faculty of Veterinary Medicine, South Valley University, Qena 83522, Egypt; mohamedmekky@vet.svu.edu.eg; 2 Laboratory of Microbial Technology, Division of Systems Bioengineering, Department of Bioscience and Biotechnology, Faculty of Agriculture, Graduate School, Kyushu University, Fukuoka 819-0395, Japan; yasaigatabetai21@yahoo.co.jp (N.I.); nakayama@agr.kyushu-u.ac.jp (J.N.)

**Keywords:** enterocin F4-9, glycocin, bacteriocin, biosynthesis, glycosylation

## Abstract

Enterocin F4-9 belongs to the glycocin family having post-translational modifications by two molecules of *N*-acetylglucosamine β-*O*-linked to Ser37 and Thr46. In this study, the biosynthetic gene cluster of enterocin F4-9 was cloned and expressed in *Enterococcus faecalis* JH2-2. Production of glycocin by the JH2-2 expression strain was confirmed by expression of the five genes. The molecular weight was greater than glycocin secreted by the wild strain, *E. faecalis* F4-9, because eight amino acids from the N-terminal leader sequence remained attached. This N-terminal extension was eliminated after treatment with the culture supernatant of strain F4-9, implying an extracellular protease from *E*. *faecalis* F4-9 cleaves the N-terminal sequence. Thus, leader sequences cleavage requires two steps: the first via the EnfT protease domain and the second via extracellular proteases. Interestingly, the long peptide, with N-terminal extension, demonstrated advanced antimicrobial activity against Gram-positive and Gram-negative bacteria. Furthermore, *enfC* was responsible for glycosylation, a necessary step prior to secretion and cleavage of the leader peptide. In addition, *enfI* was found to grant self-immunity to producer cells against enterocin F4-9. This report demonstrates specifications of the minimal gene set responsible for production of enterocin F4-9, as well as a new biosynthetic mechanism of glycocins.

## 1. Introduction

Foodborne bacteria are responsible for various outbreaks and have a significant impact on public health and the economy. Moreover, drug-resistant foodborne microbes in the current era demand intensive efforts, particularly in developing nations. Bacteriocins from lactic acid bacteria have the possibility as natural preservatives to limit the growth of undesirable bacteria in food [1].

Bacteriocins are ribosomally synthesized bacterial peptides with antimicrobial activity against other closely related bacteria. Bacteriocins display a wide range of variety in terms of molecular weight, target molecule, biosynthetic mechanism, immunity systems, antibacterial spectra, and structures [2].

Glycocins (**glyco**peptide bacterio**cins**) are bacteriocins that are subjected to post-translational modification where sugar moieties are bound to side chains of either cysteine, serine, or threonine residues. Antibacterial action is largely dependent on sugar moieties [3]. A small number of glycocins have been identified to date: sublancin 168, produced by *Bacillus subtilis* 168 [4]; glycocin F, produced by *Lactobacillus plantarum* KW30 [5]; thurandacin, encoded in *Bacillus thuringiensis* [6]; enterocin F4-9, produced by *Enterococcus faecalis* F4-9 [7]; ASM1, homologous to glycocin F produced by *Lactobacillus plantarum* [8]; and pallidocin, produced by *Aeribacillus pallidus* 8 [9]. Moreover, blast screening showed that two hypothetical peptides, Hyp1 and Hyp2, have little sequence identity to pallidocin generated in *Bacillus megaterium* BHG1.1 and *Bacillus* sp. JCM 19047 gene sequences, respectively [9].

The mechanism of action of glycocins has not been thoroughly elucidated. Sublancin 168 has a bactericidal mode of action, and the phosphoenolpyruvate: sugar phosphotransferase system plays a significant role in its activity [3,10]. However, glycocin F and enterocin F4-9 show bacteriostatic activity [5,7].

The biosynthetic mechanism of glycocin sublancin 168 is extensively studied [4]. Sublancin 168 biosynthesis is mediated by *sunA*, *sunS*, *sunT*, *bdbA*, *bdbB,* and *sunI*. *sunS* encodes *S*-glycosyltransferase, which is responsible for the glycosylation of the precursor peptide encoded by *sunA*. The protein encoded by *sunT* is expected to cleave the leader sequence and transfer the glycocin external of the producer strain. *bdbA* and *bdbB* are thought to play a role in the production of disulfide bonds in glycocin. Moreover, it has been reported that *sunI* has an immune function against mature active sublancin 168. An identical genetic grouping was discovered in the biosynthetic gene clusters for thurandacin [6] and glycocin F [5].

Enterocin F4-9 is a novel glycosylated bacteriocin purified from the culture supernatant of *Enterococcus faecalis* F4-9, isolated from Egyptian salted fish [7]. In addition to *enfA49*, which encodes the enterocin F4-9 precursor, four genes, *enfT*, *enfB*, *enfC*, and *enfI* are identified in the enterocin F4-9 gene cluster (accession no. LC029806). The database search showed that *enfC* is highly similar to a glycosyltransferase gene, the group 2 family protein of *E. faecalis* TX1346 (accession no. EFU16565), while *enfT*, *enfB*, and *enfI* were predicted to be genes encoding an ABC transporter (ATP-binding protein) (82% identity to an ABC transporter and ATP-binding protein of *E. faecalis*, accession no. WP_002396197), a thiol-disulfide isomerase that enables disulfide bond creation (38% similarity to a thioldisulfide isomerase of *Paenibacillus peoriae* (accession no. WP_010348043), and an immunity protein (66% similarity to the bacteriocin immunity protein for mundticin KS of *Enterococcus mundtii* (accession no. BAB88213), respectively [7].

In this study, the enterocin F4-9 gene cluster was characterized for functions to elucidate the biosynthetic mechanism by employing *E*. *faecalis* JH2-2 expression strains. Some heterologous expressions were conducted to determine the roles of the genes in the putative enterocin F4-9 biosynthetic gene cluster and to characterize the biosynthetic mechanism of enterocin F4-9. The constructed transformants were evaluated for production and immunity against enterocin F4-9.

## 2. Materials and Methods

### 2.1. Bacterial Strains, Plasmids, and Culture Conditions

The bacterial strains and plasmids used in this study are listed in Table 1. *E. faecalis* F4-9 and *E*. *faecalis* JH2-2 were cultivated in De Man, Rogosa and Sharpe (MRS) medium (Oxoid, Basingstoke, United Kingdom) at 30 °C for 16 h. *E. faecalis* JCM 5803^T^ was cultivated in MRS medium at 37 °C. *Proteus vulgaris* F24B was cultured in Tryptic Soy Broth (BD, Sparks, MD, USA) supplemented with 0.6% yeast extract (Nacalai Tesque, Kyoto, Japan) (TSBYE) at 37 °C, whereas *Escherichia coli* DH5α, *E. coli* JM109, and *Salmonella enterica* subsp. *enterica* NBRC 13245^T^ were cultivated in Luria–Bertani (LB) broth at 37 °C.

### 2.2. Plasmid Construction for Heterologous Expression

Plasmids and primers used in this study are listed in Table 1 and Table 2, respectively. Molecular cloning was conducted according to general protocols [13]. The KOD One TM PCR master mix (Toyobo, Osaka, Japan) and Quick Taq HS polymerase (Toyobo) were used for PCR and colony PCR, respectively, and Expin PCR SV (Gene All Biotechnology, Seoul, Korea) was used to purify DNA from PCR products or other enzymatic reaction mixtures. Enterocin F4-9 biosynthetic genes were successively cloned from two fragments into the pMG36c vector. The first fragment, encompassing the *enfT* and *enfA49* genes, was amplified via PCR using the PstI-*enfT*-f and SphI-*enfA49*-r primers. Subsequently, the PCR product was digested with PstI and SphI restriction enzymes and next ligated to the PstI- and SphI-digested pMG36c plasmid to generate the pF49-TA construct. The second fragment, which encompasses *enfB*, *enfC*, and *enfI*, was amplified using the SphI-*enfB*-f and KpnI-*enfI*-r primers. The obtained PCR product was digested with SphI and KpnI restriction enzymes and then ligated into the SphI- and KpnI-digested pF49-TA plasmid to generate the pF49-TABCI plasmid construct, which enclosed the whole region including all five genes from *enfT* to *enfI*.

The pF49-TABI plasmid, which lacks *enfC* and is assumed to play a role in the glycosylation process, was constructed. pF49-TABI was successively constructed from two fragments and cloned into the pMG36c vector. The first fragment containing the *enfT*, *enfA49*, and *enfB* genes was amplified by PCR using the PstI-*enfT*-f and SphI-*enfB*-r primers. Consequently, the PCR product was digested with PstI and SphI restriction enzymes and then ligated to the PstI- and SphI-digested pMG36c plasmid to generate the pF49-TAB construct. The second fragment containing the *enfI* gene was amplified by PCR using the SphI-*enfI*-f and KpnI-*enfI*-r primers. The obtained PCR product was digested with the SphI and KpnI restriction enzymes and then ligated to the SphI- and KpnI-digested pF49-TAB plasmid to generate the pF49-TABI plasmid construct, which enclosed the entire region, except *enfC*.

Plasmid pF49-I, containing the expected immunity gene *enfI* which is supposed to impart resistance to enterocin F4-9, was constructed. Briefly, *enfI* was amplified using the SphI-*enfI*-f and KpnI-*enfI*-r primers. The resulting PCR product was digested with SphI and KpnI restriction enzymes and subsequently ligated into the SphI-and KpnI-digested pMG36c plasmid to generate the pF49-I construct.

All plasmid constructs were introduced into *E*. *faecalis* JH2-2, an expression host. Transformations were performed by electroporation using a MicroPulser (Bio-Rad Laboratories, Hercules, CA, USA), following the method reported by Holo and Nes [14].

The ability of *E*. *faecalis* JH2-2 strains expressing the *enfA49* gene cluster to secrete and produce mature enterocin F4-9 was assessed via antibacterial activity, as described below. The immunity of transformant strains against enterocin F4-9 was assessed using an antibacterial activity assay as described below.

### 2.3. Bacteriocin-Activity Assay

The antibacterial activity of enterocin F4-9 in transformants was assessed using the spot-on-lawn assay as described previously [15], using *E*. *faecalis* JCM 5803^T^ as an indicator strain. Briefly, 10 μL of the cell-free culture supernatant of recombinant *E*. *faecalis* JH2-2 strains was spotted onto a double-layered agar plate containing 5 mL of Lactobacillus Agar AOAC medium (BD) inoculated with an overnight culture of the indicator strain as the upper layer and 10 mL of the MRS broth with 1.5% agar as the bottom layer. After overnight incubation at 37 °C, bacterial lawns were evaluated for the inhibition zone, and the minimum inhibitory concentrations (MICs) of bacteriocins toward the indicator strains were assessed. In this study, the MIC was defined as the lowest bacteriocin concentration to achieve a clear zone of growth inhibition in the spot-on-lawn assay, as in a previous study [7].

### 2.4. Purification and Determination of the Molecular Weight of Bacteriocin from the Transformants

The transformant *E*. *faecalis* JH2-2 containing pF49-TABCI was cultivated in 1 L of MRS medium at 30 °C for 16 h. The bacteriocin was purified from the culture supernatant and analyzed as described by Maky et al. [7]. Briefly, the bacteriocin was purified by adsorption onto Amberlite XAD-16 (Sigma-Aldrich, St. Louis, MO, USA) and SP Sepharose Fast Flow (Cytiva, Marlborough, MA, USA) cation exchange chromatography, and pure bacteriocin was obtained in the active fractions using reverse-phase HPLC that employed a Resource RPC 3 mL column (Cytiva). The molecular weight of the purified bacteriocin was determined by electrospray ionization time-of-flight mass spectrometry (ESI-TOF MS) using a JMS-T100LC mass spectrometer (JEOL, Tokyo, Japan).

### 2.5. Characterization of the Cleavage of the Leader Peptide

The bacteriocin secreted by the transformant strain had a longer N-terminal extension than that secreted by the original wild strain, indicating that cleavage of the leader peptide was different in the wild strain and the transformant. Effects of temperature and extracellular proteases on cleavage were characterized.

To assess the effect of temperature on leader peptide cleavage, both wild and transformant strains were cultivated in MRS at a lower temperature, i.e., 25 °C. Secreted bacteriocins were purified, and their molecular weights were analyzed as described above.

To evaluate the effect of proteolytic enzymes on the length of the N-terminal sequence, the long peptide was treated with the culture supernatant of the *E*. *faecalis* F4-9 wild strain. Briefly, *E*. *faecalis* F4-9 was incubated alone in MRS at 25 °C for 24 h, and then the culture supernatant, as a source of proteolytic enzymes, was obtained by centrifugation and filter-sterilized using DISMIC 13CP cellulose acetate filters (0.2 μm pore size; Advantec, Tokyo, Japan). Subsequently, the purified long peptide was mixed with the F4-9 culture supernatant and incubated at 25 °C for 24 h. The resulting peptide was purified from the mixture, and its molecular weight was analyzed as described above.

### 2.6. Immunity Assay

Immunity to enterocin F4-9 was assessed using the spot-on-lawn assay. Transformant *E*. *faecalis* JH2-2 containing pF49-I and transformant pF49-TABCI were inoculated in MRS supplemented with 0.8% agar and 10 μg mL^−1^ chloramphenicol and then exposed to 10 μL culture supernatants of *E*. *faecalis* F4-9. *E*. *faecalis* JH2-2 containing the empty pMG36c vector served as a negative control.

### 2.7. Functional Characterization of the Glycosyltransferase

The pF49-TABI plasmid, which lacks *enfC*, was constructed to elucidate the role of *enfC*. The ability of the recombinant *E*. *faecalis* JH2-2 strain containing pF49-TABI to produce peptides in the culture supernatant was examined by purification as described above. Purified fractions were analyzed by ESI-TOF MS to confirm the presence or absence of the peptides with the expected molecular weights. Intracellular fractions of the transformants (harboring pF49-TABI) were also analyzed. Peptides like the enterocin F4-9 precursor peptides that accumulated in transformant cells were collected as previously described [16]. Peptides in the intracellular fraction were characterized for their antimicrobial activity using the spot-on-lawn assay as described above.

## 3. Results

### 3.1. Genes Responsible for the Production and Maturation of Enterocin F4-9

**Figure 1:**
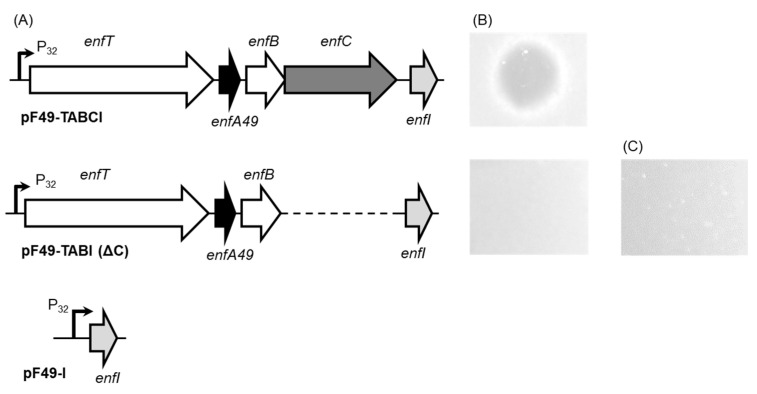
Plasmids construction for functional characterization of the enterocin F4-9 gene cluster and antibacterial activity of the resulting transformants. (**A**) Schematic illustrations of plasmids constructed in this study. Constructed plasmids were cloned and introduced into *E*. *faecalis* JH2-2, a host strain. (**B**) Evaluation of production of enterocin F4-9. Culture supernatants of transformants harboring pF49-TABCI and pF49-TABI were evaluated by the spot-on-lawn assay using *E*. *faecalis* JCM 5803^T^ as an indicator strain. (**C**) Evaluation of the intracellular fraction of the transformant harboring pF49-TABI by the spot-on-lawn assay using *E. faecalis* JCM 5803^T^ as an indicator strain.

In our attempt to identify genes responsible for the secretion and maturation of enterocin F4-9, plasmids including *enfT*, *enfA49*, *enfB*, *enfC*, and *enfI* genes were constructed utilizing the broad-host-range cloning vector pMG36c, which holds a strong constitutive lactococcal promoter P32 [11]. As a result, the secretion of mature enterocin F4-9 was detected in *E*. *faecalis* JH2-2 harboring the pF49-TABCI plasmid. Antimicrobial activity was confirmed using the spot-on-lawn method (Figure 1). This result suggested that the five genes were sufficient to produce mature and active enterocin F4-9.

Furthermore, the putative glycosyltransferase *enfC* was identified in the *enfA49* gene cluster [7]. To study its role, the pF49-TABI plasmid lacking *enfC* was constructed and introduced into *E*. *faecalis* JH2-2. The antimicrobial assay using the culture supernatant showed no activity. Furthermore, ESI-TOF MS of the purified fraction from the culture supernatant displayed no relevant peptides. These results stipulate that the glycosylation process is essential for bacteriocin secretion. Antimicrobial assays of intracellular fractions demonstrated no activity (Figure 1C). These results indicate the relevance of the glycosylation process for cleavage of the leader peptide and its antibacterial activity.

### 3.2. Purification and Mass Spectrometry of the Bacteriocin Produced by the Transformants

**Figure 2:**
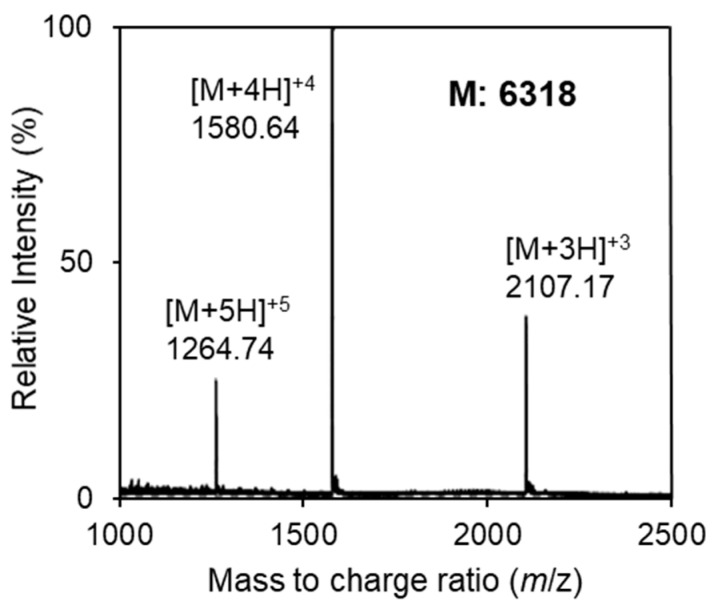
Electrospray ionization time-of-flight (ESI-TOF) mass spectrum of the purified bacteriocin from *E*. *faecalis* JH2-2 harboring pF49-TABCI (long peptide). Multiple charged molecular ions were detected and are indicated. The molecular mass of bacteriocin was calculated based on the most abundant peak.

**Figure 3:**
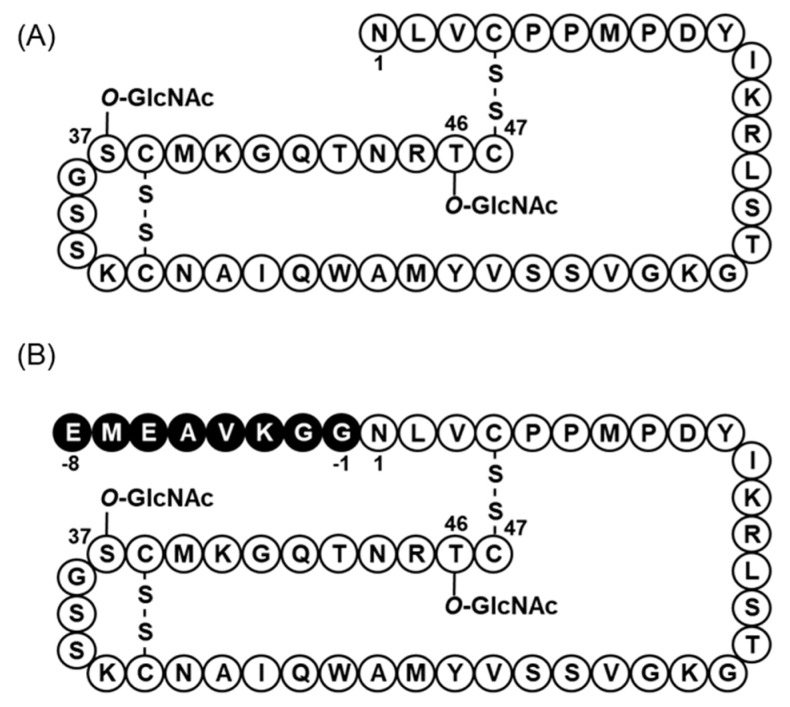
Structures of enterocin F4-9 and the long peptide. Original enterocin F4-9 (**A**) was produced by *E*. *faecalis* F4-9, and the long peptide (**B**) was produced by *E*. *faecalis* JH2-2 harboring pF49-TABCI. Eight amino acid residues indicated by black in the N-terminal represent the elongated sequence.

The bacteriocin produced by *E*. *faecalis* JH2-2 containing pF49-TABCI was purified from the culture supernatant by Amberlite XAD-16 and SP Sepharose, and finally, the pure bacteriocin was obtained as a single active peak in reverse-phase HPLC. The molecular weight of the purified bacteriocin was determined to be 6318 by ESI-TOF MS (Figure 2) indicating that mature glycosylated bacteriocin was successfully produced. However, the molecular weight of the bacteriocin produced by the wild strain is 5518 [7]. This discrepancy in the molecular weights was attributed to the eight C-terminal residues of the N-terminal leader sequence (EMEAVKGG), which was remained attached to the purified active peptide (Figure 3).

First, we hypothesized that incubation temperature may influence leader peptide cleavage [17]. To test this hypothesis, both wild and transformant strains were cultured in MRS medium at a lower temperature (25 °C); as a result, short and long peptides were detected, respectively. This finding indicated that the incubation temperature had no effect on the cleavage of the leader peptide.

**Figure 4:**
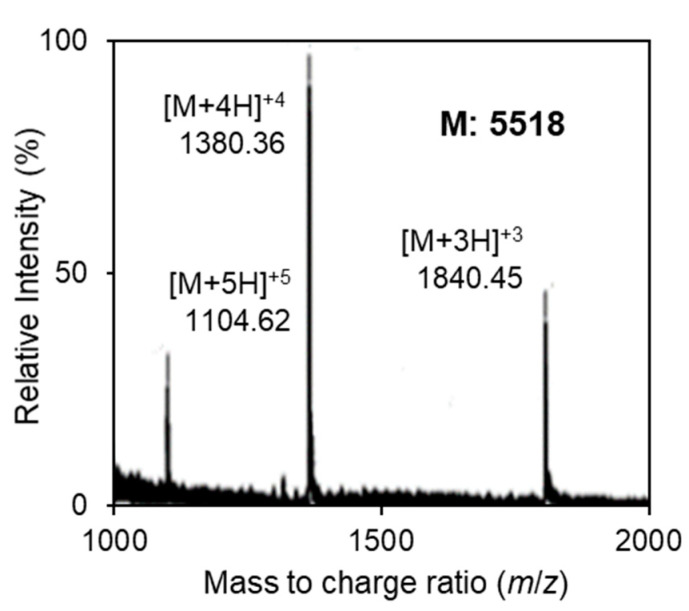
Electrospray ionization time-of-flight (ESI-TOF) mass spectrum of the long peptide treated with the culture supernatant of *E*. *faecalis* F4-9. Peptides were purified from the treated mixture and analyzed. Multiple charged molecular ions were detected and are indicated. The molecular weight of the peptide was calculated based on the most abundant peak.

The second assumption was that the possibility of the occurrence of intrinsic proteases in the wild strain can result in the second cleavage of the leader sequence. The short peptide was only produced and detected when the long peptide was incubated with the culture supernatant of the *E*. *faecalis* F4-9 wild strain (Figure 4). These data imply that lack of production of the short peptide in the transformant is related to a lack of the intrinsic protease system. Furthermore, we treated the long peptide with the culture supernatant of *E. faecalis* JH2-2 for 24 h. As a result, only the active long peptide was detected by the mass spectrum. This result confirmed that cleavage of long peptide requires a specific protease.

### 3.3. Antimicrobial Activity of Long and Short Peptides

The antimicrobial spectra of short and long peptides were compared (Table 3). The long peptide showed enhanced antimicrobial activity and a broad spectrum, in contrast to the short peptide. Interestingly, the long peptide was quite effective against *Salmonella enterica* subsp. *enterica*, whereas the short peptide had no effect. Results indicated that elongation of the N-terminal sequence enhanced antibacterial activity.

### 3.4. Characterization of the Immunity Protein for Enterocin F4-9

**Figure 5:**
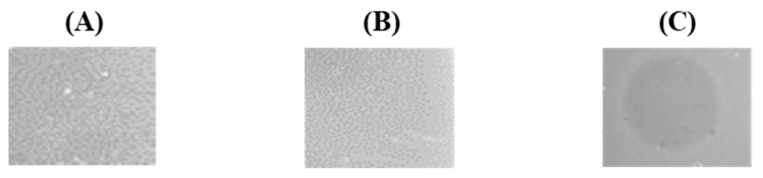
Immunity assay of *E*. *faecalis* JH2-2 transformants. *E*. *faecalis* JH2-2 transformants harboring pF49-I (**A**), pF49-TABCI (**B**), and empty pMG36c vector ((**C**), negative control) were tested for tolerance to enterocin F4-9 by the spot-on-lawn assay. The culture supernatant of *E*. *faecalis* F4-9 was used for enterocin F4-9 preparation.

The putative immunity gene, *enfI*, was detected in the *enfA49* gene cluster [7]. To characterize its role, an immunity assay was conducted with the transformants containing pF49-I or pF49-TABCI in *E*. *faecalis* JH2-2. In our results, both transformants were tolerant to the short peptide (the culture supernatant of *E. faecalis* F4-9) (Figure 5). Furthermore, both transformants were tolerant also to the long peptide (the culture supernatant of the transformant harboring pF49-TABCI) (data not shown).

## 4. Discussion

Enterocin F4-9 is a novel di-glycosylated bacteriocin discovered in Egyptian salted fish. Enterocin F4-9 has glycosylation and disulfide bond post-translational modifications; these two properties tend to be characteristics of glycocins [3]. Examples of naturally occurring glycocins are very rare, and elucidation of their biosynthesis is still ongoing. Functional characterization of the enterocin F4-9 gene cluster can help to improve the antimicrobial spectra and facilitate bioengineering for further application in food and medicine.

Here, the functional characterization of the enterocin F4-9 gene cluster was achieved, and its biosynthetic mechanism was characterized. The entire enterocin F4-9 biosynthetic gene cluster was cloned and functionally expressed in a homologous system using *E*. *faecalis* JH2-2 as the host strain. As a result, active antimicrobial peptide was obtained, indicating that the five genes, *enfT*, *enfA49*, *enfB*, *enfC*, and *enfI* collaborate in the biosynthesis, secretion, maturation, and immunity mechanism of enterocin F4-9. Notably, the biosynthesis of well-studied glycocin sublancin 168 is expressed by the gene cluster harboring *sunA*, the precursor peptide that is modified by glycosyltransferase (*sunS*), *sunT*, an ABC-transporter/peptidase, *bdbA* and *bdbB*, responsible for disulfide bond formation, and *sunI*, responsible for immune function [4]. Similarly, five genes were responsible for the biosynthesis of pallidocin, *palA*, *palS*, *palT*, *paldbA*, and *paldbB*. They were expressed in *E*. *coli* BL21 as a host strain and were sufficient for the production and secretion of pallidocin [9]. Moreover, the gene cluster of glycocin F (GccF) was identified, which includes the genes encoding GccA, a glycosyltransferase, GccB, an ABC-transporter with C39-domain, GccC, and GccD, which play a role in disulfide bond formation; GccE, a putative LytTR response regulator, and GccH and GccI, which have immunity functions [18]. The *asm* gene cluster, which encodes proteins involved in the biosynthesis of ASM1, was found to be very homologous to the glycocin F gene cluster [8].

The molecular weight of the peptide produced from the transformant strain was greater than that produced from the wild-type strain. To investigate this, the long peptide was incubated with the culture supernatant of the *E*. *faecalis* F4-9 wild strain for 24 h, resulting in the production of the short peptide. This indicated that enterocin F4-9 was processed by an extracellular protease. Alignment of glycocins showed that the leader sequences of SunA, ThuA, PalA, and Hyp1 were highly similar. In contrast, the leader sequence of EnfA49 exhibited low identity. The leader sequence is likely to play a key role in directing the processing step [19]. SunA, ThuA, Hyp2, and PalA precursors contain a Gly-Ser-Gly sequence, and the cleavage site is between the Ser and second Gly residues, whereas peptide Hyp1 has Gly-Lys-Gly sequences [9]. The other glycocins, including enterocin F4-9, have a double-glycine cleavage site.

Extracellular proteases have been found to cleave the leader sequences of some bacteriocins. In some class I bacteriocins (lantibiotics), the modified precursor peptides are exported by an ABC transporter to the extracellular space, and then the leader peptides are cut by a serine protease, resulting in the generation of mature peptides [20]. Subtilin is a type-A lantibiotic, and its maturation necessitates proteolytic cleavage of the leader peptide by three extracellular serine proteases. Furthermore, the gene cluster of subtilin has no processing protease genes, suggesting that an intrinsic protease is capable of processing the prepeptide [21]. The leader peptide cleavage of cytolysin in *E*. *faecalis*, a lantibiotic, is carried out by an ABC transporter, and the six additional amino acids are removed extracellularly by a proteolytic enzyme [22]. *E*. *faecalis* can produce various proteases such as gelatinase and serine-protease. Further work on evaluating the use of extracellular protease inhibitors could aid in identification of proteases responsible for the cleavage. The results of the current study provide evidence for the unique and novel biosynthetic mechanism of enterocin F4-9 among glycocins.

The enterocin F4-9 gene cluster contains the *enfB* gene that may be involved in the formation of disulfide bonds in enterocin F4-9 [7]. All glycocins have been found to have two structural disulfide bonds. Furthermore, one putative thioredoxin was assumed to aid in the production of proper disulfide bonds [6]. BdbA and BdbB, thiol-disulfide oxidoreductases, play a role in disulfide bond formation in sublancin 168 [23]. Breakdown of disulfide bonds resulted in the loss of the antibacterial activity of sublancin 168 [4], enterocin F4-9 [7] and pallidocin [9], demonstrating their relevance for antimicrobial activity. Based on the structural prediction, EnfB seems to be an intracellular protein that promotes the formation of disulfide bonds in the precursor peptide. However, as previously mentioned, disulfide linkages can be formed spontaneously during peptide extraction and/or purification by air oxidation [9], which makes it difficult to characterize the function of EnfB and other homologous proteins.

Generally, most bacteriocins of Gram-positive bacteria have antimicrobial activity only against Gram-positive bacteria. The outer membrane of Gram-negative bacteria reduces the activity of Gram-positive bacteriocins. The antibacterial action of the long enterocin F4-9 was considerably altered when the sequence was lengthened. It revealed activity against *Salmonella enterica* that was unaffected by the short peptide. There are little data on bacteriocins with leader sequences with antibacterial activity. Previous reports on sublancin 168 and thurandacin illustrated the need for cleavage of the leader peptide to achieve antimicrobial activity [4,6]. The synthesized pallidocin with the complete leader amino acid sequence still exhibited antimicrobial activity but was weaker than that without the leader sequence [9]. To the best of our knowledge, this is the first report of glycocins that additional amino acid residues from the leader sequence enhanced antimicrobial activity against both Gram-positive and Gram-negative foodborne pathogens.

The reason for wild-type cells to undergo secondary processing remains unknown. Long peptides may have deleterious impacts on cell stability and cell wall integrity in comparison to short peptides. Similarly, cytolysin undergoes a second proteolytic cleavage, which aids in immunity and protects the cell from its cytolytic effects [24]. However, the putative immunity protein, EnfI, conferred sufficient tolerance to both long and short peptides, suggesting that the additional sequence and/or processing may have functions other than expected.

The *enfA49* gene cluster contains a putative immunity gene, *enfI*. Transformants expressing *enfI* showed tolerance against both long and short peptides, suggesting that this single protein was sufficient to confer complete immunity to enterocin F4-9 in the host cell. The immunity protein is approximately 11 kDa in size, consisting of 98 amino acid residues, and further structural prediction suggested that it is a possible membrane protein. In the same context, a single gene (*asmH*) has been suggested to be responsible for the immunity to ASM1 [8]. Denham et al. [25] reported that *sunI* is responsible for encoding the immunity protein for sublancin 168. ThuI is a putative immunity protein detected in the thurandacin gene cluster [6]. However, in the case of glycocin F, GccH and GccI are suggested to play a role in the immune response, although their roles have not yet been studied [18].

The *enfA49* gene cluster encompasses a putative glycosyltransferase gene, *enfC*. To elucidate its function, a plasmid harboring the whole enterocin F4-9 biosynthetic gene cluster, except *enfC*, was constructed and introduced into *E*. *faecalis* JH2-2. Purification and analysis of the HPLC profile together with ESI-TOF MS confirmed that no precursor peptide was secreted extracellularly from the transformant. Similarly, it has been reported that glycosylation is required prior to glycocin F secretion [18]. Sublancin biosynthesis includes the glycosylation process regulated by SunS followed by exportation regulated by SunT [26]. These results support our findings that the precursor peptide was glycosylated before release. Furthermore, the intracellular fractions did not show any antimicrobial activity when tested using the spot-on-lawn method. A previous study reported that chemically deglycosylated enterocin F4-9 lost its antimicrobial activity [7]. Thus, genetic and chemical experiments indicated the importance of sugar moieties in the antimicrobial activity of glycocins.

Based on the findings of this study, the following biosynthetic mechanism of enterocin F4-9 can be proposed. EnfC modifies the precursor peptide, pre-enterocin F4-9, encoded in *enfA49*, to generate the glycosylated peptide. After the glycosylation process, EnfT transports the peptide, where the cleavage of peptides requires a two-step process involving cleavage by the protease domain in EnfT followed by removal of eight additional amino acids by extracellular proteases. EnfB assists with disulfide bridge formation in the peptide, probably within cells prior to secretion, as in the case of sublancin [4]. The findings demonstrate that *E*. *faecalis* F4-9 has a unique biosynthetic mechanism. Our findings provide fresh insights into the bioengineering of bacteriocins, which have immense potential for use in food and medicine.

## Figures and Tables

**Table 1:** Bacterial strains and plasmids used in this work.

Strain or Plasmid	Description	Source and/or Reference
Strains		
*E. faecalis*		
F4-9	Enterocin F4-9 producer strain	[7]
JH2-2	Plasmid-free derivative of *E*. *faecalis* JH-2	[11]
JCM 5803^T^	Indicator strain sensitive to enterocin F4-9	JCM
*Escherichia coli*		
DH5α	Plasmid storage strain	Takara
JM109	Indicator strain sensitive to enterocin F4-9	JCM
*Salmonella enterica* subsp. *enterica* NBRC 13245^T^	Indicator strain for enterocin F4-9	NBRC
*Proteus vulgaris* F24B	Indicator strain for enterocin F4-9, isolated and identified from Japanese fish.	This study
Plasmids		
pMG36c	Cm^r^, pWV01-based cloning vector carrying a strong *Lactococcus*-based promoter, P_32_	[12]
pF49-TA	Cm^r^, pMG36c derivative containing *enfT* and *enfA49*	This study
pF49-TABCI	Cm^r^, pMG36c derivative containing *enfT*, *enfA49*, *enfB*, *enfC* and *enfI*	This study
pF49-I	Cm^r^, pMG36c derivative containing *enfI*	This study
pF49-TABI	Cm^r^, pMG36c derivative containing *enfT*, *enfA49*, *enfB* and *enfI*	This study

Cm^r^, chloramphenicol resistant. JCM, Japan Collection of Microorganisms (Wako, Japan). NBRC, National Institute of Technology and Evaluation Biological Resource Center (Chiba, Japan).

**Table 2:** Primers used in this study.

Primer	Nucleotide Sequence (5′-3′) ^a^	Purpose
PstI-*enfT*-f	AACTGCAGGAAATACATCAGTATGG	Amplification of *enfT*-*A*
SphI-*enfA49*-r	AAGCATGCGAATTGAAAATTCAGCC	Amplification of *enfT*-*A*
SphI-*enfB*-f	AAGCATGCGAATAGATACTAGGAG	Amplification of *enfB*-*C*-*I*
KpnI-*enfI*-r	AAGGTACCCTAAATCGAGGAG	Amplification of *enfB*-*C*-*I*
SphI-*enfI*-f	AAGCATGCCATTAAGGAGGCT	Amplification of *enfI*
KpnI-*enfI*-r	AAGGTACCCTAAATCGAGGAG	Amplification of *enfI*
SphI-*enfB*-r	AAGCATGCCGAGTATCCTAAAA	Amplification of *enfT-B*

^a^ Restriction sites are underlined.

**Table 3:** Antimicrobial spectrum of the purified short and long peptides.

Indicator Strain	MIC (μM) ^a^	
	Short Peptide	Long Peptide
*Enterococcus faecalis* JCM 5803^T^	1.87	0.93
*Escherichia coli* JM109	1.87	0.46
*Salmonella enterica* subsp. *enterica* NBRC 13245^T^	NA	0.23
*Proteus vulgaris* F24B	3.74	0.23

^a^ NA, no activity (>30 μM).

## Data Availability

Data sharing not applicable.
